# Overexpression of B7H5/CD28H is associated with worse survival in human gastric cancer

**DOI:** 10.1111/jcmm.14812

**Published:** 2019-12-28

**Authors:** Can Hu, Zhiyuan Xu, Shangqi Chen, Hang Lv, Yiping Wang, Xiaofeng Wang, Shaowei Mo, Chengwei Shi, Shenyu Wei, Liqiang Hu, Wei Chen, Xiangdong Cheng

**Affiliations:** ^1^ First Clinical Medical College Zhejiang Chinese Medical University Hangzhou Zhejiang China; ^2^ Department of Abdominal Surgery Zhejiang Cancer Hospital Hangzhou Zhejiang China; ^3^ Key Laboratory of Integrated Traditional Chinese and Western Medicine for Diagnosis and Treatment of Digestive System Tumor Hangzhou Zhejiang China; ^4^ Cancer Institute of Integrated Traditional Chinese and Western Medicine Key Laboratory of Cancer Prevention and Therapy Combining Traditional Chinese and Western Medicine Zhejiang Academy of Traditional Chinese Medicine Hangzhou Zhejiang China

**Keywords:** B7 family checkpoints, B7H5, CD28H, gastric cancer, immunotherapy

## Abstract

Gastric cancer (GC) is a common malignancy with low 5‐year overall survival (OS). Recently, immune therapy has been used to treat cancer. B7H5 and CD28H are novel immune checkpoint molecules. However, the prognostic value of B7H5/CD28H expression in patients with GC remains unclear. In this study, seventy‐one patients diagnosed with GC were included in this study. Patients' GC tissues and matched adjacent tissue constructed a tissue microarray. The expression levels of B7H5 and CD28H were examined using immunohistochemistry. Correlations between the expression of B7H5 and CD28H and the clinical data were evaluated. We found that the expression of B7H5 and CD28H (both *P* = .001) were higher in GC tumour tissues than in adjacent noncancerous tissues. B7H5/CD28H expression acted as an independent predictive factor in the OS of patients with GC. High expression of B7H5 and CD28H predicted poor outcome. Patients in the B7H5+CD28H+ group had a lower 5‐year OS compared with patients in the B7H5−CD28− group (4.5% vs 55.6%, *P* = .001). A significant difference was found in the 5‐year OS between patients in the B7H5+CD28H− and B7H5+CD28H+ groups (33.5% vs 4.5%, *P* = .006). However, there was no correlation between B7H5 and CD28H expression (*P* = .844). Therefore, B7H5 and CD28H expression are up‐regulated in GC and are independent prognostic factors for overall survival in patients with GC. Although there was no correlation between B7H5 and CD28H expression, high expression of B7H5 and CD28H predicts poor prognosis, especially when both are highly expressed.

## 1 INTRODUCTION

Gastric cancer (GC) is a common malignancy and has been estimated to account for one‐third of cancer deaths.^1^ Surgery is the optimal treatment for patients with GC, which provides the best chance of long‐term survival. However, the early stage of the disease is often asymptomatic, and GC is frequently diagnosed in the later stages, when it is characterized by invasion or metastasis. The therapeutic options of neoadjuvant therapy, targeted drugs and immunotherapy have opened a new field of cancer treatment in recent years; however, the 5‐year overall survival (OS) rate of GC is unsatisfactory.^2^ While cancers suppress the immune response by establishing a microenvironment, the immune cells decrease cancer immunogenicity in the process of tumorigenesis and cancer progression.^3^ Therefore, the interaction between the microenvironment and the immune system is a new direction in GC research.^4^ However, patients with GC have not benefited from immunotherapy.

Immune checkpoint therapy is a novel treatment that targets regulatory pathways in T cells to enhance antitumour immune responses. The B7/CD28 family of ligands and receptors play an important role in cancer pathogenesis. Ten members are included in the B7/CD28 family, including B7‐1/CD28, B7‐2/cytotoxic T lymphocyte antigen 4 (CTLA‐4), ICOS‐L/ICOS and programmed cell death 1 (PD‐1)/PD‐1 ligand (PD‐L1).^5^ PD‐L1/PD‐1 has become a hot area of research in recently years.^6^, ^7^ The B7/CD28 family regulates the proliferation and function of T cells as antigen‐presenting cells (APCs). In addition, some tumours can evade immune elimination, because of overexpression of PD‐L1/PD‐1.^8^, ^9^ Inhibitors of PD‐L1/PD‐1 have been approved by Food and Drug Administration (FDA) as second‐line treatments for lung cancer, because of their therapeutic benefit in clinical trials.

The B7H5/CD28H pathway is a novel receptor‐ligand interaction in the B7/CD28 family that can reduce the expression of interleukin‐5 (IL‐5), IL‐13, IL‐10 and tumour necrosis factor gamma (TNF‐γ) and suppress the activity of CD4+ and CD8+ T cells.^10^ Some studies showed low expression of B7H5 in normal tissue,^11^ while B7H5 is overexpressed in some cancers.^12^, ^13^ However, the prognostic value of B7H5/CD28H in patients with GC is controversial. In the present study, we aimed to evaluate the expression levels of B7H5 and CD28H in patients with GC.

## 2 MATERIALS AND METHODS

### 2.1 Tissue microarray (TMA) construction and immunohistochemistry (IHC) analysis

Seventy‐one formalin‐fixed, paraffin‐embedded (FFPE) GC tissues and corresponding adjacent noncancerous tissues were collected from the Department of Abdominal Surgery, Zhejiang Cancer Hospital. All FFPE GC tissues were screened by two pathologists independently to confirm the diagnosis of GC. The most representative tumour and noncancerous tissues were selected to construct the TMA slide. Seventy‐one paired GC tissues and matched noncancerous tissues were included in the TMA.

The IHC technique has been described in detail previously.^14^ TMA sections were deparaffinized, and hydrogen peroxide (3%) was applied to repair the antigen. Nonspecific staining was blocked using 10% goat serum at room temperature for 30 minutes. Immunostaining of histological sections was performed using monoclonal antibodies against B7H5 and CD28H (Gifts from Professor Yuwen Zhu, University of Colorado Anschutz Medical Campus) overnight at 4°C followed by a 30‐minutes incubation with goat anti‐rabbit IgG (H&L) Biotin secondary antibody (dilution 1:1,000; cat. no AB97049; BioVision, Inc)/goat anti‐mouse IgG (H&L) Biotin secondary antibody (dilution 1:500; cat. no AB97049; BioVision, Inc) and visualization with 3,3′‐diaminobenzidine (DAB) for 3 minutes. Harris haematoxylin was used to stain the nuclei. The sections were left to dehydrate and were then sealed with neutral gel. Categorization of the scanned images was performed using density quant software in the Quant Center (3DHistech, Ltd).

Cytoplasmic expression was assessed using the H‐score system. The formula for the H‐score was as follows: H‐score = ∑ (IS × AP), where IS represents the staining intensity and AP represents the percentage of positively stained tumour cells, producing a cytoplasmic score ranging between 0 and 12. An IS between 0 and 3 was assigned for the intensity of tumour cell staining (0, no staining; 1, weak staining; 2, intermediate staining; 3, strong staining). AP depended on the percentage of positive‐stained cells as follows: 0 (0%), 1 (1%‐25%), 2 (26%‐50%), 3 (51%‐75%) and 4 (75%‐100%). The score was assigned using the estimated proportion of positively stained tumour cells. To assess the average degree of staining within a tumour sample, multiple regions were analysed, and at least 100 tumour cells were assessed. Two assessors who were blinded to the clinical outcomes performed the scoring independently.

### 2.2 Flow cytometry

Ten fresh GC tissues and their corresponding adjacent noncancerous tissues were collected from the Department of Gastrointestinal Surgery, The First Affiliated Hospital of Zhejiang Chinese Medical University. Single‐cell suspensions from the tissues were stained for extracellular surface antigens using the anti‐CD28H monoclonal antibodies, followed by incubation with goat anti‐mouse IgG Biotin secondary antibody, FITC anti‐human CD3 antibody and 7‐AAD Viability staining solution. Finally, viable single cells were analysed.

### 2.3 Post‐operative evaluation and follow‐up

Post‐operative chemotherapy was performed according to each patient's individual pathological diagnosis. If an infiltrating margin was found, adjuvant irradiation was necessary. The patients were examined regularly every 3 months during the first year and every 6 months thereafter.

### 2.4 Statistical analysis

Statistical analysis was performed using IBM SPSS statistics for Mac, version 23.0 (IBM Corp). Survival curves were estimated using the Kaplan‐Meier method, and multivariate and univariate analyses were performed using a Cox stepwise proportional hazard model. A *P*‐value <.05 was considered statistically significant.

## 3 RESULTS

### 3.1 Patient characteristics

Among the 71 patients with GC, 53 (74.65%) were male and 18 (23.35%) were female, with a median age of 67 (range 34‐83) years. At the end of follow‐up, 47 (66.20%) patients had died and 24 (33.80%) patients were alive. According to the depth of tumour invasion, 4 (5.63%) were at T1, 6 (8.45%) were at T2, 47 (66.20%) were at T3, and 14 (19.72%) were at T4. For lymph node metastasis, 19 (26.76%) were N0, 15 (21.13%) were N1, 21 (29.58%) were N2, and 16 (22.54%) were N3. For distant metastasis, 68 (95.77%) were M0 and 3 (4.23%) were M1. According to the 8th AJCC staging system, 7 (9.86%) were at stage I, 25 (35.21%) were stage at II, 35 (47.95%) were at stage III, and 4 (5.64%) were at stage IV (Table 1).

**Table 1:** Correlation between B7H5 expression and clinicopathological characteristics

Variables	B7H5 expression		Total	χ^2^	*P*‐value
	High	Low			
Age (y)					
≤66	26	9	35	0.039	.844
>66	16	20	36		
T stage					
T1/T2	4	6	10	4.141	**.042** a
T3/T4	47	14	61		
TNM stage					
Ι/II	25	7	32	0.709	.400
III/IV	27	12	39		
N stage					
N0	17	2	19	3.488	.062
N1/N2/N3	35	17	52		
M stage					
M0	49	18	67	0.000	1.000
M1	3	1	4		
P53					
Negative	36	12	48	0.010	.922
Positive	17	6	23		
Sex					
Female	14	4	18	0.038	.845
Male	38	15	53		
Ki67					
Negative	18	14	32	8.579	**.003** a
Positive	34	5	39		
Grade					
I/II	20	3	28	3.266	.071
III/IV	32	16	53		

a Statistically significant (*P* < .05).

### 3.2 B7H5 expression in cytoplasm in GC and noncancerous tissues

**Figure 1:**
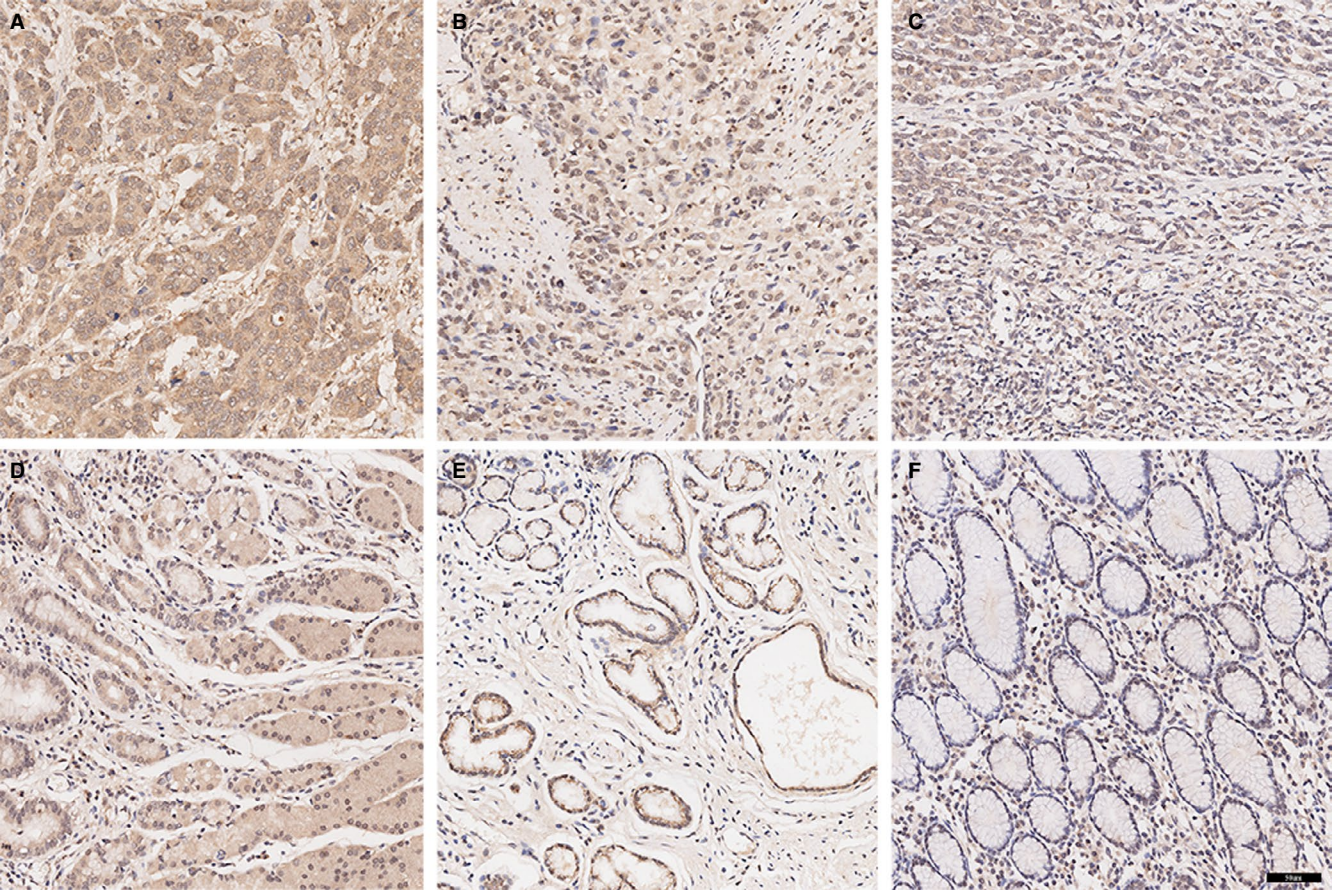
Expression of B7H5 in gastric carcinoma and noncancerous tissues. A‐C, High/middle/low expression of B7H5 in gastric carcinoma. D‐F, High/middle/low expression of B7H5 in noncancerous tissues

**Figure 2:**
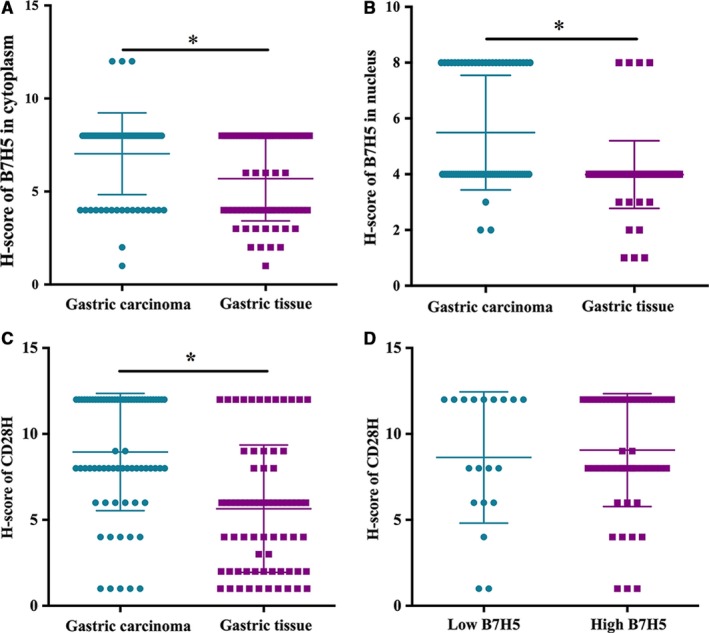
B7H5 protein was expressed in both gastric carcinoma and noncancerous tissues. A, B7H5 expression was higher in cytoplasm in GC tissues than in gastric tissue (*P* = .001); B, B7H5 expression was higher in the nucleus in GC tissues than in gastric tissue (*P* < .001); C, CD28h expression was higher in immune cells in GC tissues than in gastric tissue (*P* = .001); D, There was no correlation between B7H5 and CD28H expression (*P* = .844)

B7H5 protein levels in GC tissues are currently unknown. As shown in Figure 1, B7H5 was expressed in the cytoplasm in the noncancerous and GC tissues. However, the staining intensity of B7H5 in the cytoplasm in GC tissues was obviously stronger than that in noncancerous tissues (Figure 2A). The median H‐score of B7H5 was 8 (range: 1‐12). The median score was used to determine the cut‐off value of low‐ or high‐level B7H5 expression. An H‐score <8.0 was defined as low B7H5 expression, and an H‐score ≥8.0 was defined as high B7H5 expression. Nineteen (26.76%) patients showed low B7H5 expression, while 52 (73.24%) showed high B7H5 expression in GC tissues. However, 32 (45.07%) showed low B7H5 expression, and 39 (54.93%) patients showed high B7H5 expression in noncancerous tissues (*P* = .001).

### 3.3 B7H5 expression in the nucleus in GC and noncancerous tissues

B7H5 was expressed in the nucleus in the noncancerous tissues and GC tissues. However, the staining intensity of B7H5 in the nuclei in GC tissues was obviously stronger than that in noncancerous tissues (Figure 2B). Forty‐four (61.97%) patients showed low B7H5 expression, while 27 (38.03%) showed high B7H5 expression in GC tissues. However, 67 (94.37%) showed low B7H5 expression, and 4 (5.63%) showed high B7H5 expression in noncancerous tissues (*P* < .001).

### 3.4 CD28H^+^ T cells exist in GC and noncancerous tissues

**Figure 3:**
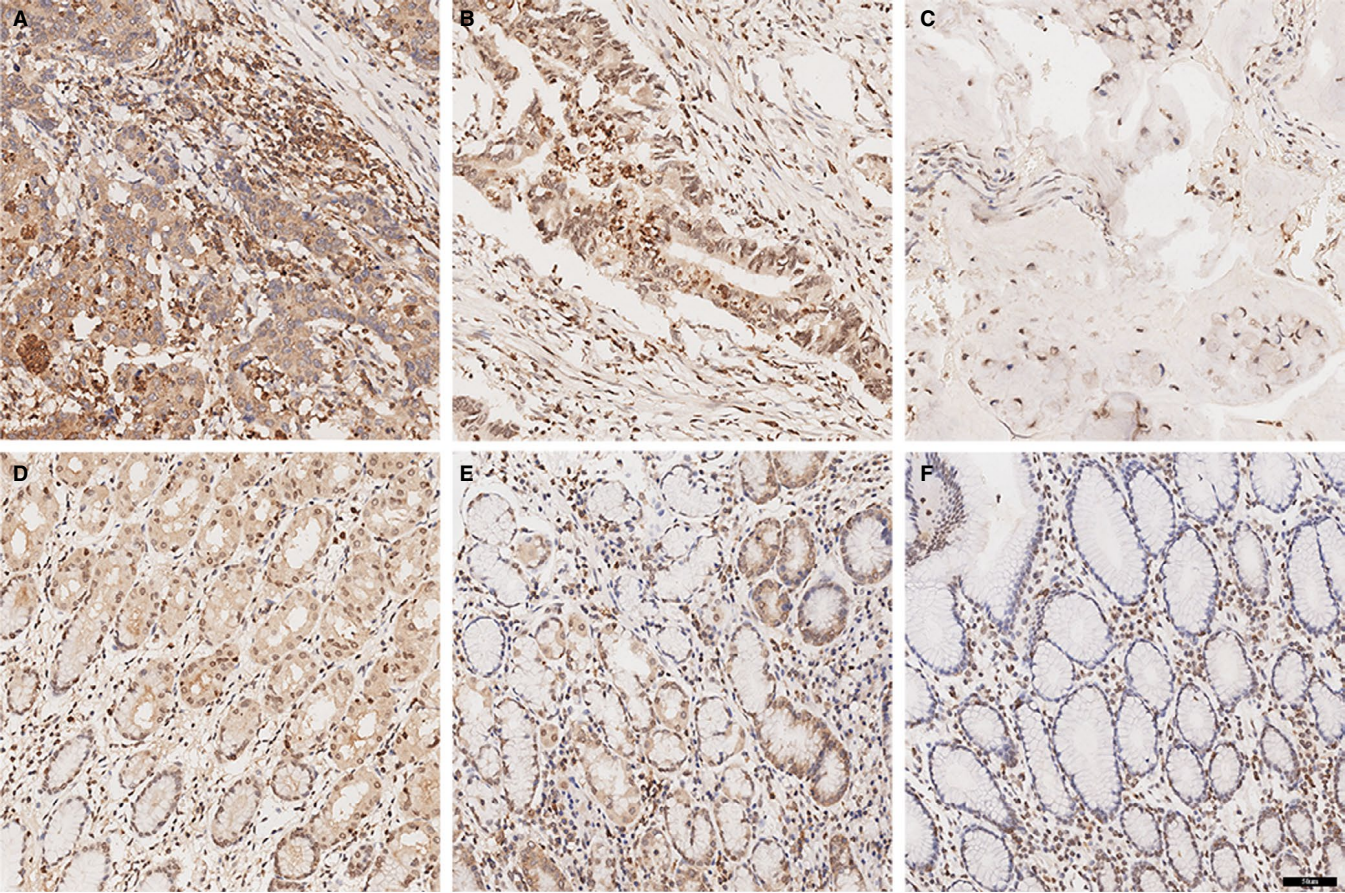
Expression of CD28H in gastric carcinoma and noncancerous tissues. A‐C, High/middle/low expression of CD28H in gastric carcinoma. D‐F, High/middle/low expression of CD28H in noncancerous tissues

**Figure 4:**
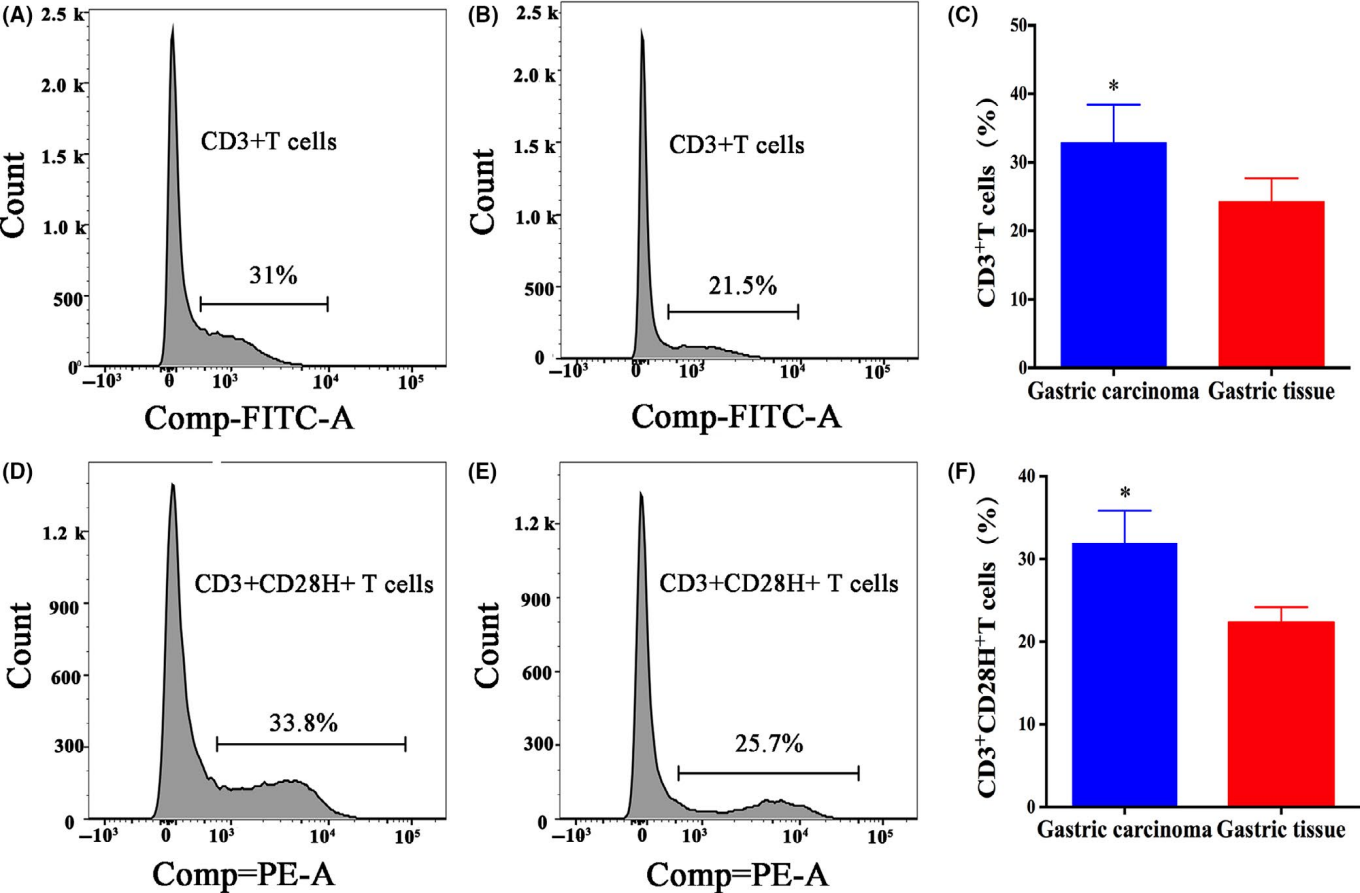
CD28H is expression on T cells in GC tissues and gastric tissues. A‐B, Representative flow cytometry plots showing the count of CD3^+^T cells in GC tissues and gastric tissues. C, The mean percentages showed that the count of CD3^+^T cells in GC tissues was higher than that than gastric tissues, *, *P* < .05. D‐E, Representative flow cytometry plots showing the count of CD3^+^CD28H^+^T cells in CD3^+^ T cells in GC tissues and gastric tissues. F, The mean percentages showed that the count of CD3^+^ CD28H^+^T cells in CD3^+^ T cells in GC tissues was higher than that in gastric tissues, *, *P* < .05

CD28H protein levels in GC tissues are currently unknown. As shown in Figure 3, the CD28H protein was expressed in both GC and noncancerous tissues. In the GC tissues and noncancerous tissues, CD28H was expressed in immune cells. However, the staining intensity of CD28H in GC tissues was obviously stronger than that in noncancerous tissues. The median of H‐score of CD28H was 8 (range: 1‐12). The median score was used to determine the cut‐off value of low or high CD28H expression. An H‐score ≤8 was defined as low CD28H expression, and an H‐score >8 was defined as high CD28H expression. Thirty‐six (50.70%) patients showed low CD28H expression, while 35 (49.30%) showed high CD28H expression in GC tissues. However, 17 (23.94%) showed low CD28H expression, while 54 (76.06%) showed high CD28H expression in noncancerous tissues (*P* = .001). In addition, we analysed CD28H expression on CD3^+^T cells in GC tissues and gastric tissues using flow cytometry. As shown in Figure 4, the level of CD3^+^T cells in GC tissues was higher than that in gastric tissues (*P* < .01), while the level of CD3^+^CD28H^+^T cells was higher in GC than in gastric tissues (*P* < .01).

### 3.5 B7H5 and CD28H expression in GC and clinicopathological variables

The association between B7H5 and CD28H expression and clinicopathological parameters in GC was investigated using the chi‐square test. As listed in Table 1, a significant correlation was found between the depth of invasion (T) (*P* = .042) and Ki67 expression (*P* = .003), while B7H5 expression in the cytoplasm was not significantly associated with age, sex, lymph node metastasis (N), distant metastasis (M), TNM stage, p53 expression or pathological grade. However, as shown in Tables 2 and 3, B7H5 expression in the nucleus and CD28H expression in immune cells was not significantly associated with age, sex, T stage, N stage, M stage, TNM stage, Ki 67 stage, p53 expression or pathological grade.

**Table 2:** Correlation between B7H5 expression in the nucleus and clinicopathological characteristics

Variables	B7H5 expression		Total	χ^2^	*P*‐value
	High	Low			
Age (y)					
≤66	12	23	35	0.410	.522
>66	15	21	36		
T stage					
T1/T2	3	7	10	0.045	.831
T3/T4	24	37	61		
TNM stage					
Ι/II	15	17	32	1.935	.164
III/IV	12	27	39		
N stage					
N0	7	12	19	0.015	.901
N1/N2/N3	20	32	52		
M stage					
M0	26	41	67	0.001	.982
M1	1	3	4		
P53					
Negative	10	38	48	0.787	.375
Positive	7	16	23		
Sex					
Female	8	10	18	0.421	.516
Male	19	34	53		
Ki67					
Negative	11	21	32	0.330	.566
Positive	16	23	39		
Grade					
I/II	10	18	28	0.109	.741
III/IV	17	36	53		

Statistically significant (*P* < .05).

**Table 3:** Differential expression of CD28H in cancerous and gastric tissues

Variables	CD28H expression		Total	χ2	*P*‐value
	High	Low			
Age (y)					
≤66	16	19	35	0.354	.552
>66	19	17	36		
T stage					
T1/T2	3	7	10	0.952	.329
T3/T4	32	29	61		
TNM stage					
Ι/II	13	19	32	0.063	.801
III/IV	22	17	39		
N stage					
N0	7	12	19	1.610	.205
N+	28	24	52		
M stage					
M0	35	32	67	2.296	.130
M1	0	4	4		
P53					
Negative	30	18	48	0.233	.630
Positive	13	10	23		
Sex					
Female	12	6	18	2.911	.088
Male	23	30	53		
Ki67					
Negative	15	19	32	0.700	.403
Positive	20	17	39		
Grade					
I/II	9	14	23	1.406	.236
III/IV	26	22	49		

Statistically significant (*P* < .05).

### 3.6 Prognostic value of B7H5 and CD28H expression in patients with GC

**Figure 5:**
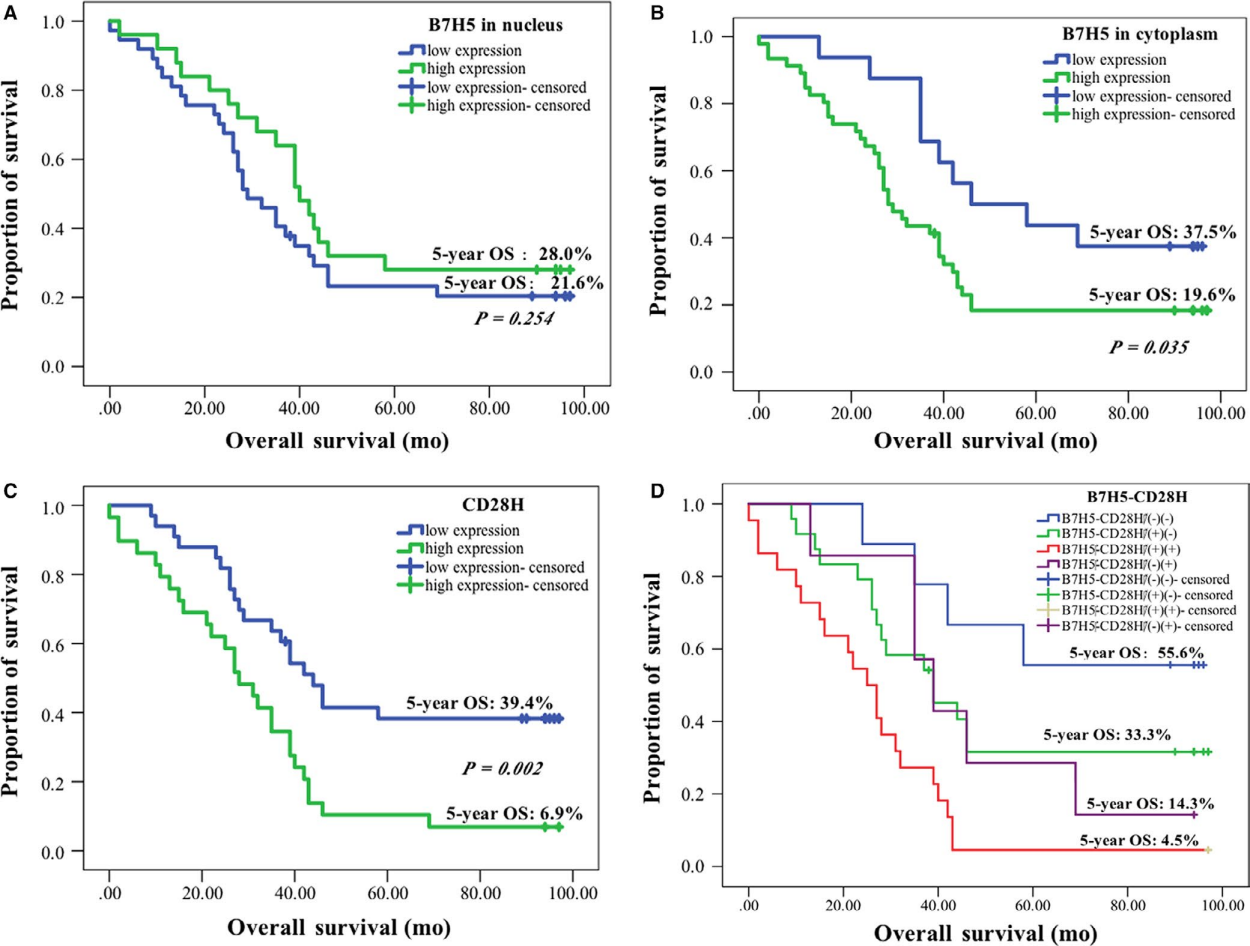
Correlation of B7H5/CD28H expression level and overall survival (OS) in GC patients. A, There is no difference in 5‐year OS between high B7H5 expression and low expression in the nuclei in patients with GC (28.0% vs 21.6%, *P* = .254); B, High B7H5 expression predicted poorer survival than low B7H5 expression in patients with GC (19.6% vs 37.5%, *P* = .035); C, High CD28H expression predicted poorer survival than low CD28H expression in patients with GC (39.4% vs 6.9%, *P* = .002); D, Patients in the B7H5^+^CD28H^+^ group have a lower 5‐year OS compared with patients in the B7H5^−^CD28^−^ group (4.5% vs 55.6%, *P* = .001), a significant difference was found in the 5‐year OS between patients in B7H5^+^CD28H^−^ and B7H5^+^CD28H^+^ groups (33.5% vs 4.5%, *P* = .006), and there was no significant difference between the B7H5^−^CD28^−^ group and the B7H5^−^CD28H^+^ group (55.6% vs 33.3%, *P* = .111)

As shown in Figure 5, the prognostic value of B7H5 expression in GC was investigated. Although there was no difference in 5‐year OS between high B7H5 expression and low expression in the nucleus among patients with GC (28.0% vs 21.6%, *P* = .254), survival analysis showed that a high cytoplasmic B7H5 expression predicted poorer survival than a low cytoplasmic B7H5 expression in patients with GC (19.6% vs 37.5%, *P* = .035), while GC patients with high CD28H had poorer survival (39.4% vs 6.9%, *P* = .002). According to the expression of B7H5 in the cytoplasm and CD28H in immune cells, all patients were divided into four group, including low B7H5 and CD28H expressions (B7H5^−^CD28H^−^) group, low B7H5 expression with high CD28H expression (B7H5^−^CD28H^+^) group, high B7H5 expression with low CD28H expression (B7H5^+^CD28H^−^) group, and high B7H5 and CD28H expression (B7H5^+^CD28H^+^) group. The 5‐year OS was 4.5% in the B7H5^+^CD28H^+^ group, 14.3% in the B7H5^−^CD28H^+^ group, 33.3% in the B7H5^+^CD28H^−^ group and 55.6% in the B7H5^−^CD28H^−^ group. There was a significant difference between B7H5^+^CD28H^+^ patients and B7H5^−^CD28H^−^ patients (*P* = .001), while there was no difference between B7H5^−^CD28H^−^ patients and B7H5^−^CD28H^+^ patients (*P* = .111), or between B7H5^+^CD28H^−^ and B7H5^−^CD28H^−^patients (*P* = .173). There was a significant difference between B7H5^+^CD28H^−^ and B7H5^+^CD28H^+^ patients (*P* = .006), while there is no difference between B7H5^+^CD28H^−^ and B7H5^−^CD28H^+^ patients (*P* = .777). In addition, there was no difference between B7H5^−^CD28H^+^ and B7H5^+^CD28H^+^ patients (*P* = .066). However, the results of the chi‐square test confirmed that there was no correlation between B7H5 expression and CD28H expression in patients with GC (*P* = .844). As listed in Table 4, significant prognostic factors for survival by univariate analysis were T stage, N stage, AJCC stage, B7H5 expression and CD28H expression. Multivariate analysis using the Cox proportional hazard model confirmed that B7H5 and CD28H expression levels were independent risk factors.

**Table 4:** Prognostic factors in univariate and multivariate analyses

Factors	Univariate analysis *P*‐value	Multivariate analysis	
		*P*‐value	HR (95%Cl)
Gender			
Male vs Female	.747	.581	1.247 (0.570‐2.726)
Age			
<67 vs ≤67	.326	.287	1.396 (0.756‐2.578)
B7H5 expression			
Low vs High	.035^a^	.016^a^	2.575 (1.196‐5.544)
CD28H expression			
Low vs High	.002^a^	.017^a^	2.337 (1.167‐4.682)
T stage			
T2 vs T3, T4	.003^a^	.192	2.758 (0.601‐12.666)
N stage			
N0 vs N1, N2	.024^a^	.426	1.561 (0.522‐4.664)
M stage			
M0 vs M1	.273	.493	1.604 (0.415‐6.199)
AJCC stage			
I, II vs III, IV	.001^a^	.174	1.956 (0.744‐5.141)
Pathological grade			
I, II vs III, IV	.887	.244	0.651 (0.316‐1.340)

Abbreviations: CI, confidence interval; HR, hazard ratio.

a Statistically significant (*P* < .05).

## 4 DISCUSSION

The present study aimed to explore the clinical significance of the B7H5/CD28H pathway in GC and confirmed that the activities of B7H5 and CD28H might represent a new immunosuppressive mechanism within the GC microenvironment, as well as providing novel targets for GC immunotherapy. In the present study, we reported that B7H5 was overexpressed both in the cytoplasm and nucleus in tumour tissues from patients with GC compared with that in the adjacent noncancerous tissues. In addition, there was no difference in 5‐year OS between high B7H5 expression and low expression in the nucleus in patients with GC (28.0% vs 21.6%, *P* = .254), while high cytoplasmic B7H5 expression predicted poorer survival than did low cytoplasmic B7H5 expression in patients with GC (19.6% *vs*. 37.5%, *P* = .035). A study by Zhao et al showed that B7H5 was expressed in the cytoplasm of mouse embryonic fibroblast cells and could reduce the production of cytokines from T cells.^15^ This suggested that B7H5 expression in the cytoplasm might be important in immunotherapy. This was the same as the results of previous studies showing that B7H5 was expressed in the oesophagus, liver, colon and pancreas.^12^, ^16^ However, a previous study by Shimonosono^17^ compared B7H5 expression in peripheral blood between 111 patients with untreated GC and 20 healthy volunteers. Their study showed that patients with GC had significantly higher *B7H5* mRNA levels and higher B7H5 expression was associated with a better 5‐year OS. This result revealed a different B7H5 expression pattern to that shown in the present study. We are unable to explain this difference because of the different methods and antibodies used to detect B7H5 expression between the two studies. However, we also showed that the expression of B7H5 was almost absent in B7H5^KO^‐BGC803 group in vivo. And it can also indicate the specificity of B7H5 (as showed in Figure S1). Other studies confirmed that high B7H5 expression was associated with poor prognosis in certain tumours. Janakiram et al^18^ showed that overexpression of B7H5 was associated with advanced stage of the disease and predicted high recurrent risk in breast cancer. In addition, Koirala et al^19^ confirmed that B7H5 was expressed in human osteosarcoma and was associated with metastases and worse survival. Our study also confirmed that B7H5 expression correlation with Ki67 expression in patients with GC (*P* = .003); Ki67 expression was detected in patients with GC with high B7H5 expression. Ki67 is an antigen associated with proliferation, and overexpression of Ki67 is negatively correlated with carcinoma differentiation.^20^, ^21^ It further revealed that high B7H5 expression predicted poor outcome in patients with GC.

B7H5 has two receptors on T cells, including CD28H and another, as yet unknown, receptor. B7H5 has co‐stimulatory and co‐inhibitory effects against the immune response of T cells by CD28H and the unknown receptor.^10^ Therefore, we also examined the expression of CD28H. We found that the level of CD28H^+^ T cells in the tumour tissues in patients with GC was higher than that in the adjacent noncancerous tissues. Furthermore, patients in the B7H5^+^CD28H^+^ group had a lower 5‐year OS compared with patients in the B7H5^−^CD28H^−^ group (*P* = .001). However, there was no significant difference between the B7H5^−^CD28H^−^group and the B7H5^−^CD28H^+^ group (*P* = .111), while a significant difference was found in the 5‐year OS between patients in B7H5^+^CD28H^−^ and B7H5^+^CD28H^+^ groups (*P* = .006). The results revealed that high expression of B7H5 and CD28H predict poor prognosis, especially when both are highly expressed, because of inhibition of the immune response of T cells. Moreover, B7H5 and CD28H acted as independent predictive factors in the overall survival of patients with GC. However, there was no correlation between B7H5 and CD28H expression (*P* = .844).

Our study showed that the B7H5/CD28H axis is a significant predictor of poor outcome. However, a new study by Yan et al showed that B7H5 is overexpressed in pancreatic ductal adenocarcinoma (PDAC) and high B7H5 expression is associated with better survival.^22^ Therefore, this study reminds us that different molecular mechanisms of B7H5 exist in different tumours. Some members of the B7/CD28 family have two opposing effects in different immune microenviroments.^23^, ^24^ For example, B7H3 has a T‐cell co‐stimulatory and a co‐inhibitory role in the immune response, 25, 26, 27similar to B7H5. B7H5 and CD28H are new members of the B7/CD28 family. The interaction between B7H5 and CD28H can promote the proliferation and cytokine production of T cells via the AKT pathway, while some studies confirmed that B7H5 could prevent the expression and secretion of cytokines by T cells to inhibit their response, including the IL‐5, IL‐10, IFNγ and TNFα.^10^ Therefore, the interaction of B7H5 and CD28H may inhibit the immune response as a co‐inhibitor in GC.

In conclusion, we confirmed that B7H5 and CD28H expression levels are up‐regulated and predict low survival in patients with GC, and are independent prognostic factors of overall survival. Although there is no correlation between B7H5 and CD28H expression, high expression of B7H5 and CD28H predicts poor prognosis, especially when both are highly expressed, via inhibition of the immune response of T cells. Therefore, the B7H5/CD28H axis could be an attractive target for GC immunotherapy.

## CONFLICT OF INTEREST

The authors report no conflict of interest.

## AUTHOR CONTRIBUTIONS

Xiangdong Cheng and Wei Chen contributed to conception or design of the work; Can Hu and Zhiyuan Xu contributed to drafting the work; Can Hu, Zhiyuan Xu, Shangqi Chen, Shaowei Mo, Chengwei Shi, Shenyu Wei, Liqiang Hu and Xiaofeng Wang contributed to data acquisition; Hang Lv and Yiping Wang contributed to data analysis; Xiang‐dong Cheng and Can Hu contributed to supervision or mentorship. All the authors contributed important intellectual content for the overall work. Xiang‐dong Cheng, Wei Chen and Zhi‐yuan Xu take responsibility for the honesty and accuracy of the present study.

## ETHICAL APPROVAL

The study was approved by the ethics committee of Zhejiang Cancer Hospital. The study conformed to the tenets of the Declaration of Helsinki. All patients provided written informed consent before taking part in the study.

## Data Availability

The data used to support the findings of this study are available from the corresponding author upon request.

## References

[1] Hu C , Zhu HT , Xu ZY , et al. Novel abdominal approach for dissection of advanced type II/III adenocarcinoma of the esophagogastric junction: a new surgical option. J Int Med Res. 2019;47(1):398‐410. 30296865 10.1177/0300060518802923 PMC6384491

[2] Shen L , Shan YS , Hu HM , et al. Management of gastric cancer in Asia: resource‐stratified guidelines. Lancet Oncol. 2013;14:e535‐e547. 24176572 10.1016/S1470-2045(13)70436-4

[3] Chen DS , Mellman I . Oncology meets immunology: the cancer‐immunity cycle. Immunity. 2013;39:1‐10. 23890059 10.1016/j.immuni.2013.07.012

[4] Aroldi F , Zaniboni A . Immunotherapy for pancreatic cancer: present and future. Immunotherapy. 2017;9:607‐616. 28595517 10.2217/imt-2016-0142

[5] Ni L , Dong C . New B7 family checkpoints in human cancers. Mol Cancer Ther. 2017;16:1203‐1211. 28679835 10.1158/1535-7163.MCT-16-0761 PMC5568666

[6] Soyano AE , Dholaria B , Marin‐Acevedo JA , et al. Peripheral blood biomarkers correlate with outcomes in advanced non‐small cell lung Cancer patients treated with anti‐PD‐1 antibodies. J Immunother Cancer. 2018;6:129. 30470260 10.1186/s40425-018-0447-2 PMC6251165

[7] Brahmer JR , Tykodi SS , Chow LQ , et al. Safety and activity of anti‐PD‐L1 antibody in patients with advanced cancer. N Engl J Med. 2012;366:2455‐2465. 22658128 10.1056/NEJMoa1200694 PMC3563263

[8] Niemeijer AN , Leung D , Huisman MC , et al. Whole body PD‐1 and PD‐L1 positron emission tomography in patients with non‐small‐cell lung cancer. Nat Commun. 2018;9:4664. 30405135 10.1038/s41467-018-07131-y PMC6220188

[9] Oliva M , Spreafico A , Taberna M , et al. Immune biomarkers of response to immune‐checkpoint inhibitors in head and neck squamous cell carcinoma. Ann Oncol. 2019;30(1):57‐67. 30462163 10.1093/annonc/mdy507 PMC6336003

[10] Zhu Y , Yao S , Iliopoulou BP , et al. B7‐H5 costimulates human T cells via CD28H. Nat Commun. 2013;4:2043. 23784006 10.1038/ncomms3043 PMC3698612

[11] Byers JT , Paniccia A , Kaplan J , et al. Expression of the novel costimulatory molecule B7–H5 in pancreatic cancer. Ann Surg Oncol. 2015;22(suppl 3):S1574‐S1579. 25519928 10.1245/s10434-014-4293-2

[12] Zhu Z , Dong W . Overexpression of HHLA2, a member of the B7 family, is associated with worse survival in human colorectal carcinoma. Onco Targets Ther. 2018;11:1563‐1570. 29593422 10.2147/OTT.S160493 PMC5865557

[13] Chen D , Chen W , Xu Y , et al. Upregulated immune checkpoint HHLA2 in clear cell renal cell carcinoma: a novel prognostic biomarker and potential therapeutic target. J Med Genet. 2019;56(1):43‐49. 29967134 10.1136/jmedgenet-2018-105454

[14] Teng F , Xu Z , Chen J , et al. DUSP1 induces apatinib resistance by activating the MAPK pathway in gastric cancer. Oncol Rep. 2018;40:1203‐1222. 29956792 10.3892/or.2018.6520 PMC6072387

[15] Zhao R , Chinai JM , Buhl S , et al. HHLA2 is a member of the B7 family and inhibits human CD4 and CD8 T‐cell function. Proc Natl Acad Sci U S A. 2013;110:9879‐9884. 23716685 10.1073/pnas.1303524110 PMC3683785

[16] Chen J , Li XL , Zhao CK , et al. Conventional ultrasound, immunohistochemical factors and BRAF(V600E) mutation in predicting central cervical lymph node metastasis of papillary thyroid carcinoma. Ultrasound Med Biol. 2018;44(11):2296‐2306. 30100099 10.1016/j.ultrasmedbio.2018.06.020

[17] Shimonosono M , Arigami T , Yanagita S , et al. The association of human endogenous retrovirus‐H long terminal repeat‐associating protein 2 (HHLA2) expression with gastric cancer prognosis. Oncotarget. 2018;9:22069‐22078. 29774123 10.18632/oncotarget.25179 PMC5955131

[18] Janakiram M , Pareek V , Cheng H , et al. Immune checkpoint blockade in human cancer therapy: lung cancer and hematologic malignancies. Immunotherapy. 2016;8:809‐819. 27349980 10.2217/imt-2016-0001 PMC5619054

[19] Koirala P , Roth ME , Gill J , et al. HHLA2, a member of the B7 family, is expressed in human osteosarcoma and is associated with metastases and worse survival. Scientific Rep. 2016;6:31154. 10.1038/srep31154 PMC4987662 27531281

[20] Boger C , Behrens HM , Rocken C . Ki67–An unsuitable marker of gastric cancer prognosis unmasks intratumoral heterogeneity. J Surg Oncol. 2016;113:46‐54. 26709194 10.1002/jso.24104 PMC4736456

[21] Abdel‐Aziz A , Ahmed RA , Ibrahiem AT . Expression of pRb, Ki67 and HER 2/neu in gastric carcinomas: Relation to different histopathological grades and stages. Ann Diagn Pathol. 2017;30:1‐7. 28965621 10.1016/j.anndiagpath.2017.05.003

[22] Yan H , Qiu W , Koehne de Gonzalez AK , et al. HHLA2 is a novel immune checkpoint protein in pancreatic ductal adenocarcinoma and predicts post‐surgical survival. Cancer Lett. 2018;442:333‐340. 30447255 10.1016/j.canlet.2018.11.007 PMC6357962

[23] Wu D , Tang R , Qi Q , et al. Five functional polymorphisms of B7/CD28 co‐signaling molecules alter susceptibility to colorectal cancer. Cell Immunol. 2015;293:41‐48. 25497975 10.1016/j.cellimm.2014.11.006

[24] Janakiram M , Shah UA , Liu W , et al. The third group of the B7‐CD28 immune checkpoint family: HHLA2, TMIGD2, B7x, and B7–H3. Immunological Rev. 2017;276:26‐39. 10.1111/imr.12521 PMC5338461 28258693

[25] Dong P , Xiong Y , Yue J , et al. B7H3 as a promoter of metastasis and promising therapeutic target. Front Oncol. 2018;8:264. 30035102 10.3389/fonc.2018.00264 PMC6043641

[26] Wu J , Wang F , Liu X , et al. Correlation of IDH1 and B7H3 expression with prognosis of CRC patients. Eur J Surg Oncol. 2018;44:1254‐1260. 29871819 10.1016/j.ejso.2018.05.005

[27] Seaman S , Zhu Z , Saha S , et al. Eradication of tumors through simultaneous ablation of CD276/B7‐H3‐positive tumor cells and tumor vasculature. Cancer Cell. 2017;31(501–15):e8. 10.1016/j.ccell.2017.03.005 PMC5458750 28399408

