# Supplementary material for: Overexpression of B7H5/CD28H is associated with worse survival in human gastric cancer
Source: J Cell Mol Med. 2019 Dec 28;24(2):1360–9. doi: 10.1111/jcmm.14812 (PMC6991633; doi:10.1111/jcmm.14812)
Supplement: Supplementary file 1 [file JCMM-24-1360-s001.docx]

***Establishment of*** ***nude mouse tumor model***

The GC cell line BGC823 was cultured in RPMI-1640 medium (Gibco, Massachusetts, USA) with 10% fetal bovine serum (FBS; Gibco, Massachusetts, USA). The cells were cultured at 37℃ with 5% CO_2_. B7H5^KO^-BGC823 human cells line was established using the CRISPR/Cas9 lentivirus by Shanghai GenePharma company. Nude Mice were randomly divided into two group of 6 animals (NS group and B7H5^KO^ group). MGC823 cells or B7H5KO-MGC823 cells were subcutaneously injected into the right flank of nude mice at a dose of 2×107 cells per mouse in 100 µl PBS. And the tumor nodules had grown to about 1 cm for 4 weeks. Then the mice were killed and the tumor tissues was collected for immunohistochemistry staining.

***The expression of B7H5 was almost absent in B7H5KO-BGC823 group in vivo***

B7H5 was expressed in the cytoplasm and nucleus in NC group and B7H5^KO^-BGC823 group. However, the cells positive for dark brown-yellow B7H5 protein satin in the cytoplasm and nucleus were much more less in B7H5^KO^-BGC823 group than that in NC group (Fig 6).


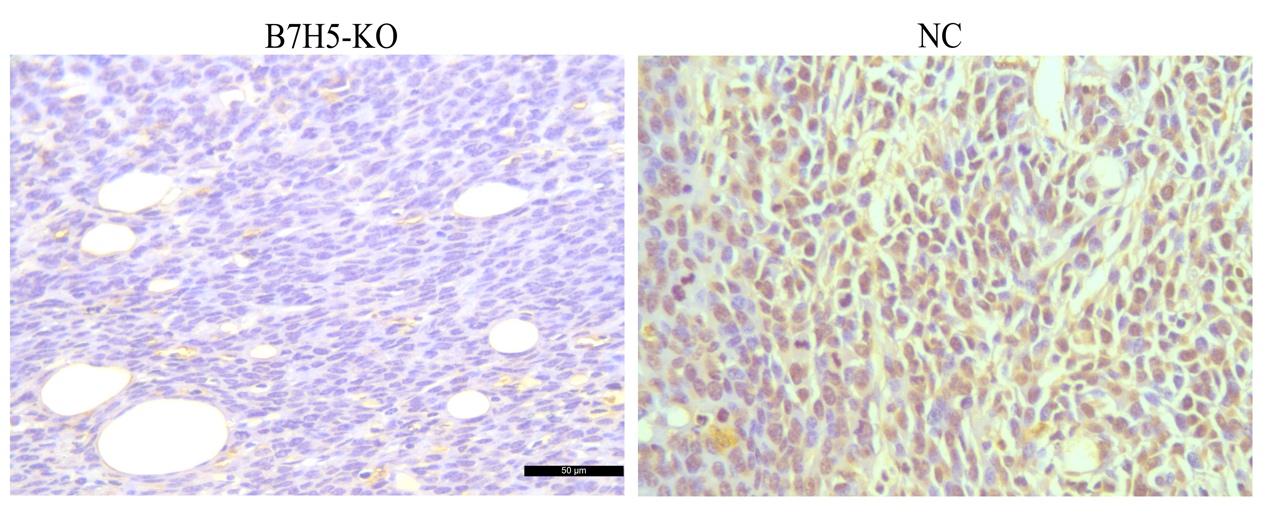


**Fig. S1** Immunocytochemistry of B7H5 protein (stained dark brown-yellow) in tumor tissues of mice in B7H5KO-BGC823 group and NC group.
